# Alleviating isolation stress in chickens: The benefits of home pen playback and mirrors

**DOI:** 10.1371/journal.pone.0318126

**Published:** 2025-02-12

**Authors:** Janja Sirovnik

**Affiliations:** Centre for Animal Nutrition and Welfare, Clinical Department for Farm Animals and Food System Science, University of Veterinary Medicine, Vienna, Austria; Chapman University, UNITED STATES OF AMERICA

## Abstract

This study investigated whether the provision of (1) a mirror or (2) video and sound playback of conspecifics in the home pen (i.e., playback) could alleviate stress in socially isolated adult domestic chickens. Thirty adult chickens participated in the study, undergoing three-minute sessions of social isolation over three consecutive days in an arena containing a tray of food treats. Each chicken was exposed to three conditions, one per day, in a semi-randomised order: (1) mirror, (2) playback, and (3) control. Video recordings of the tests were coded for stress-related behaviours, including stress behaviour (i.e., pooled stress vocalisations and escape behaviour), vigilance, feeding, and exploration. Additionally, thermal imaging was used to measure the surface temperatures of the eye and comb. Social isolation elicited mild stress responses, as evidenced by reduced surface eye and comb temperatures along with the exhibition of stress and vigilance behaviours. Both playback and mirror conditions appeared to reduce stress behaviours compared to the control, although the effect of the mirror was not statistically significant. It is possible that the playbacks simulated the presence of a group of calm conspecifics. Vigilance behaviour, however, remained unaffected across conditions. These findings suggest that playback, and to a lesser extent mirrors, may alleviate certain stress-related behaviours in socially isolated adult chickens. As individual variation was high, future research should explore the role of individual differences in stress responses, as well as the long-term effects of repeated exposure to mirrors and playback, along with other environmental variables.

## Introduction

Social separation is a common strategy for achieving standardised conditions during training and testing in social animals. To mitigate stress during these processes, animals are typically subjected to days or weeks of habituation to being separated from their peers (e.g., Campbell et al., 2019; de Haas et al., 2017 [1,2]). However, habituation is not possible in certain conditions where extensive handling prior to testing may affect the test outcome, or when animals are not marked for individual recognition, such as chickens in commercial housing systems.

Chickens are social animals and exhibit indicators of stress when separated from their peers [3–5]. These indicators include escape attempts, high pitched stress vocalisations, heightened vigilance, reduced exploration, as well as lower likelihood to feed, longer latency to feed, and less time spent feeding [2,6–8]. While stress is known to cause a decrease in surface temperature [9], its relationship to social isolation has not yet been studied. Social behaviour and motivation in chickens develop during the first weeks of life. While some stress behaviour such as vocalisations when separated from their peers decrease over time, others, like vigilance, are more prominently observed in older chickens [3,10].

The presence of a mirror has been shown to reduce stress-related behaviour in socially separated young chickens; i.e., at 1 week of age [11]. However, no studies have explored the effects of mirror presentation on stress in isolated adult chickens. In young chickens, video recordings of other chickens, with or without accompanying audio playback, have been shown to elicit an approach response, although their effects on stress-related behaviours have not been studied [12]. The combination of audio and video playback, reflecting behaviours typical of a home pen (e.g., contentment behaviour), has also not been investigated for its potential to reduce stress in chickens. Nevertheless, the combination of audio and video playback appears to be more effective in providing a sense of companionship to adult chickens than either stimulus alone [13,14].

Due to potential imprinting on the presented chickens in the video playback and mirrors [15,16], as well as other changes during ontogeny, such as decreased social reinstatement behaviour with increasing age [3,10], findings from studies on young chickens may not fully extrapolate to adult chickens. Adults may exhibit a diminished responsiveness to social stimuli during isolation.

This study aimed to investigate whether the provision of a (1) mirror or (2) video and sound playback of the conspecifics in the home pen (henceforth referred to as ‘playback’) can reduce stress in socially isolated adult chickens. It was hypothesised that adult chickens would display reduced isolation stress when presented with a mirror or playback compared to the control condition.

## Materials and methods

### Ethical statement

The study was approved by the Austrian Ministry for Education, Science and Research (GZ 2023-0.430.966). All study procedures and manuscript development were performed with regard to PREPARE and ARRIVE guidelines [17,18]. Data was collected at the Campus of the University of Veterinary Medicine, Vienna, Austria, in October 2023. No animal was sacrificed and no analgesia or anaesthesia were used in this study.

### Animals and husbandry

The study was conducted in October in 2023. One week prior to its commencement, 30 Lohmann Brown adult female chickens – the most common hybrid used for egg production in Austria – were transferred from a commercial facility to the university campus and housed in a single experimental pen. Before their arrival to the campus, the chickens had been kept in a free-range facility equipped with an aviary, adhering to Austrian regulations, with a stocking density of 9 chickens per square metre. At the time of the study, the chickens were 65 weeks old, marking the end of their production period – a stage at animal welfare assessments are commonly conducted [19,20]. A one-week acclimatisation period was deemed sufficient for adult chickens to adapt to a new social structure [21].

The housing conditions exceeded the minimum requirements for chicken housing as stipulated by Austrian regulations. The unheated home pen, measuring 19.42 m² in total, comprised two sections: an indoor area and an outdoor wintergarden (refer toS1 and S2 Fig in the supplementary material). Indoors, the chickens were provided with five nest boxes, perches, five nipple drinkers, two feeders, and wood shavings for bedding. The wintergarden featured a roof and wire mesh walls, with the lower half covered by opaque plastic panels and the upper half exposed to outdoor climatic conditions. Natural light was provided through a 97 cm W x 148 cm H window. The wintergarden was further enriched with perches, 50 cm-high opaque vertical panels, bark mulch, and two bell drinkers. Outside of the social isolation tests, the chickens had undisturbed access to both the indoor and outdoor areas of the home pen. During social isolation tests, however, they were confined to the indoor area.

### Test arena

A metal pen (100 cm L × 100 cm W × 100 cm H), located in the outdoor wintergarden, was used for social isolation testing. The walls and floor of the test arena were lined with tarpaulin, and the roof was covered with a soft-net mesh. A start box, measuring 37 cm L × 37 cm W × 34 cm H, was placed in the left corner adjacent to the door of the arena. The start box featured a handle, which was used to lift it out of the test arena at the start of the test. To ensure motivation to feed, a food tray containing highly valuable food treats, including mealworms, other insects and seeds [22] was positioned diagonally in the opposite corner of the test arena from the start box during testing. A camcorder was mounted above the arena to ensure complete visibility of the arena in the recordings.

### Experimental conditions

Three experimental conditions were used in the study: playback, mirror, and control. Each chicken was exposed to all three conditions. The objects placed in the test arena varied depending on the experimental condition. For the playback condition, the chickens had visual contact with the computer screen (Samsung Odyssey, G5, S32AG520PU, resolution: 2560 × 1440) positioned approximately 40 cm from the wall opposite the entrance. A 4 mm thick transparent plexiglass barrier separated the chickens from the screen, preventing physical access while allowing unobstructed visual access. In the mirror condition, a mirror was placed in front of the computer screen and angled to enable the chickens to see their own reflection. In the control condition, the mirror remained in the same location but angled so the chickens could only see the mirror’s backside.

The playback consisted of a video and audio recording of chickens used in the social isolation test. The recordings were made inside the wintergarden prior to testing. For these recordings, the experimenter entered the pen and remained there for approximately 30 minutes to allow the chickens to habituate to the presence of the person and the equipment. A camera (Panasonic LUMIX GH5, resolution: 3840 × 2160, 25 fps) was then used to record the chickens for approximately 10 minutes. A 30-second segment depicting chickens dustbathing, wing stretching, and foraging was selected and looped to create the playback. The playback can be accessed using this link: https://youtu.be/1Q_6iURs-c0. The playback was presented at a volume approximating the natural loudness of real chickens and remained consistent across all playback conditions for all individuals.

A food tray (40 cm × 10 cm) was placed in front of the objects, positioned diagonally from the start box, requiring the chickens to cross the test arena to access it (S3 Fig in the supplementary material). Owing to their nearly panoramic vision [23], the chickens were able to peck at the feed treats while simultaneously observing the objects.

### Habituation

Before the social isolation test, chickens were habituated to the food trays and the feed treats provided. The habituation period spanned seven days, beginning on the day the chickens arrived to the campus and continuing until the day before the social isolation testing. Two treat trays were used during this period. One tray, filled with a mixture of feed treats including mealworms, crickets, black soldier fly larvae, rye, oats, lentils, sunflower seeds, sesame seeds, flaxseed, barley, quinoa, buckwheat, and wheat, was placed in the indoor section of the pen near the feeder. The other tray was positioned in the wintergarden, adjacent to the test arena.

### Social isolation testing

The chickens were tested over three consecutive days (approximately 10:00 AM to 03:00 PM), with each chicken exposed to a different experimental condition each day. The testing order was randomised by dividing the chickens into five groups of six, with each experimental condition being randomly allocated to two chickens within each group. Additionally, the daily testing order for the groups was randomised so that each group was tested at a different time of day. The sequence in which the chickens were tested within each group was also randomised daily. During testing, the chickens were unable to hear other chickens in the indoor section of the home-pen, although they could hear the parrots and pigeons housed in neighbouring pens, which could also be heard in the audio playback.

Before each test, the chicken was brought to the wintergarden. Since reduced surface temperature (e.g., comb and eye temperatures) is a validated indicator of stress in chickens [9], a single thermal image of the chicken’s head was taken using a FLIR T650sc thermal camera (equipped with a 25° lens, resolution: 640 x 480 pixels, temperature range: −40° to 2000° C). Following this, the chicken was placed in the start box inside the test arena. After five seconds, the start box was removed through the top of the test arena by temporarily moving the mesh-net to the side. Once the mesh-net was returned to its original position covering the arena, the test commenced.

The chicken was left in the arena for 180 seconds, during which its behaviour was video recorded using the camcorder positioned above the test arena. At the end of the test, a thermal image of the chicken’s right eye was taken again with the thermal camera before the chicken was returned to the indoor section of the home pen. To minimize any influence on the temperature measurement, the chickens were handled with garden gloves before and after the test.

### Video coding

All behaviours were analysed retrospectively from the video recordings by a single observer, who was blinded to the treatments, using Solomon software version 19.08.02. The behaviours of interest and their descriptions are listed in Table 1. Stress-indicative behaviours of interest include stress vocalisations, jumps, head-out-of-arena, and vigilance. Chickens exhibited head-out-of-arena behaviour five times in control and three times in playback conditions (total n = 8) and jumps four times in the control, three times in the playback and once in the mirror condition (total n = 8). With one exception, head-out-of-arena and jumping were exhibited by the same individuals under the same conditions, and these behaviours were accompanied by stress vocalisations. Consequently, the number head-out-of-arena behaviour and jumps were pooled together with stress vocalisations into a single variable termed ‘stress behaviour’. Intra-rater reliability of all behaviours was assessed on 15% (n = 14) of all videos.

**Table 1:** Recorded behaviours and their description (adapted from [24,25]).

Behaviour	Description
Vigilance (s)	The total duration of all bouts of vigilant behaviour defined as no forwards movements, with the front of the body shifted upwards and the neck stretched upwards. The vigilant bout started with a chicken lifting the neck and it ended when the neck was lowered.
Jump (No.) ^¥^	Being in an upright position and flapping the wings rapidly until lifting off the ground.
Head-out-of-arena (No.) ^¥^	Placing the head outside the test arena, either through the mesh-net above or between the metal pole of the test arena and a tarpaulin on the side.
Stress vocalisation (No.) ^¥^	Short, high-pitch and -volume vocalisations, often occurring at the end of a bout of lower-pitch and -volume vocalisations.
Exploration (s)	Pecking (rapid touching of the beak on the ground or arena features) or ground scratching (moving foot in a rapid backwards motion against the ground). Ground scratching is often intermittent during bouts of pecking and often followed by one or two steps after ground scratch. Exploration bouts begin when the beak or front of the foot first contact the ground, and end after three seconds have elapsed without pecking or ground scratching. Pecking the feed treats and object are not considered as exploration.
Feeding duration (s)*	Pecking at the feed treats in the food tray. Pecking bouts begin when the beak first contacts the feed treats and end after three seconds have elapsed without pecking.
Latency to feed (s)	Time from the beginning of the test (i.e., the start box fully lifted) until the beak first touches the feed treats.
Latency to object (s)	Time from the beginning of the test until the chicken touches the object (mirror - front, screen, mirror-back).

¥Jumps, head-out-of-arena, and stress vocalisations were pooled into a single variable called ‘stress behaviour’.

*From the feeding duration data, it was noted, whether chickens ingested feed and coded a binary variable called feed ingestion.

### Thermal image analysis

**Fig 1:**
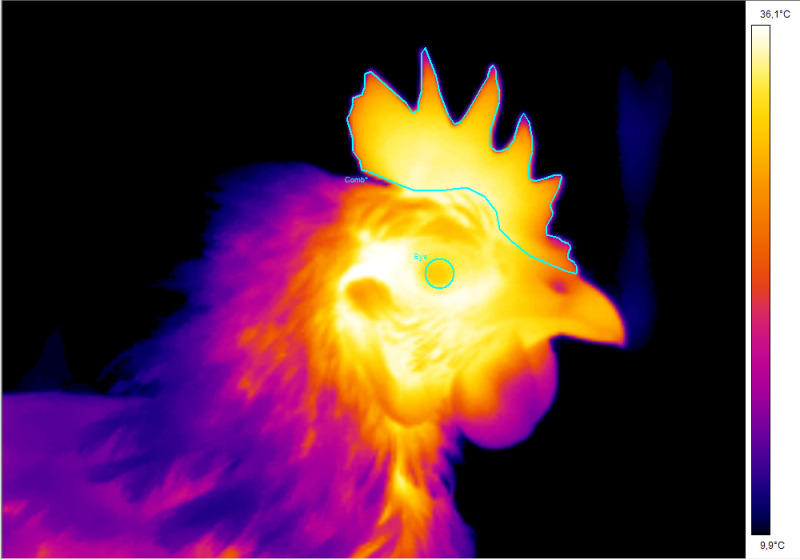
Thermal image of a domestic chicken, illustrating the measurement regions for the eye and comb.

The comb and right eye temperatures were measured from thermal images using the software package ThermaCAM Researcher Professional 2.10. The environmental temperature, consistent across both the indoor and outdoor sections of the home pen (13° Celsius), was factored in during the surface temperature calculations. The distance and emissivity were set to 1 m and 0.96, respectively. The measurement regions are shown in Fig 1. In chickens, the temperature across the head and comb can vary. Therefore, the maximum comb temperature was determined by outlining the entire comb with polygons, and the maximum eye temperature was identified by drawing a circle around the eye.

### Statistical analysis

Statistical analysis was performed in R (version 4.3.0, R Core Team, 2021) [26]. Intra-rater reliability was assessed using Intra-Class Correlation (ICC) estimates [package irr (version 0.84.1; function icc)] [27]. As a general guide, the ICC reliability coefficients were considered poor when below 0.50, moderate when between 0.50 and 0.74, good when between 0.75 and 0.89, and excellent when higher than or equal to 0.9 [28].

The data on the effects on feed ingestion, stress behaviour, and exploration were dichotomised (i.e., whether it was observed or not) and analysed using separate binomial logistic regression mixed effects models [package *lme4*, version 1.1-35.1, function *glmer*, options ‘family = binomial(link = “logit”)’] [29]. Additionally, the likelihood to express vigilant behaviour for at least 30 seconds was also analysed with a binomial logistic regression mixed effects model. Based on a visual observation of the distribution, a 30-second cut-off was used for vigilant behaviour because all chickens except four expressed some level of vigilance, but the data were right skewed.

Latencies to feed and touch objects were analysed using Cox proportional hazards regression models [package *survival*, version 3.5-5, functions *coxme and Surv*] [30]. The duration of feeding was transformed into fraction using the formula [duration feed/180)]. To avoid 0% and 100% fractions, the following transformation was performed: ((duration feed in fraction) × (length of the dataset – 1)) + 0.5)/length of the dataset [31]. The transformed data on feeding duration was analysed using a beta regression mixed-effects model [package glmmTMB, version 1.1.9, function glmmTMB, options ‘family = beta_family(link = “logit”)] [32].

Differences in the maximum surface temperature (after test - before test) of the eye and comb were analysed in separate general linear regression mixed models [package *lme4*, version 1.1-35.1, function *lmer*, option ‘REML = FALSE’]. The difference in maximum temperature was calculated by subtracting the temperature before the test from the temperature after the test for the eye and comb separately. To identify an effect of the tests on the general difference in the maximum surface temperature, models with a sum to zero restriction were used (i.e., contrast sum for the condition and test day). To compare the outcomes for the fixed effects, general linear regression mixed models without a sum to zero restriction were fitted (i.e., contrast treatment for the condition and test day).

In all models, except for the effects of feed ingestion, condition with three levels (i.e., playback, mirror, control) and test day with three levels (days 1−3) were considered as fixed effects and subject (i.e., chicken, N = 30) a random effect. To avoid model conversion problems, for the effect on feed ingestion, test day was included as covariate.

For all mixed-effects models, model stability was assessed by sequentially dropping the levels of grouping factors (i.e., subjects one at a time), fitting the full model to each subset, and then comparing the range of estimates retrieved with those obtained for the full dataset. This process revealed estimates of moderate to good stability. Multicollinearity was evaluated using variance-inflation factors [package *car*, version 3.1.2, function *vif*] [33]. For all linear mixed models, the assumptions about the residuals and random effects were visually evaluated. For the Beta regression model, assumptions about the residuals were evaluated via a simulation-based approach (package DHARMa, version 0.4.6, function simulateResiduals, option ‘n = 1000’) [34]. The beta regression model was tested for overdispersion using custom function overdisp.test, which revealed that the model was not overdispersed (dispersion parameter: 0.933). No obvious violations were observed in any of the models.

To avoid ’cryptic multiple testing’ and obtain an overall effect of condition and test day in the binomial logistic regression mixed effects model [35], the significance of individual fixed effects was determined by means of likelihood ratio tests [36] comparing the full model with models lacking them one at a time (function drop1, options ‘test = Chisq’). For the beta regression mixed effects model and Cox proportional hazards regression models, full model was compared with a null model lacking condition and test day in the fixed effects part but being otherwise identical. For all the analyses, significance was declared at an alpha cut-off of 5%. For any significant effect of condition and/or test day, post-hoc analysis [package *emmeans*, version 1.10.0, function *emmeans*, options ‘pairwise ∼ condition (or test day), adjust = “holm”, type = “response”’] [37] was performed to determine which contrasts were significant, with p-values Bonferroni-Holm-corrected for multiple testing across all contrasts. The Kaplan-Meier plot was created using the package ggfortify [version 0.4.16, functions *autoplot* and *survfit*] [38].

## Results

The coefficients for intra-rater reliability revealed excellent reliability for all indicators. Detailed results of the intra-rater reliability are provided in S1 Table in supplementary materials.

**Fig 2:**
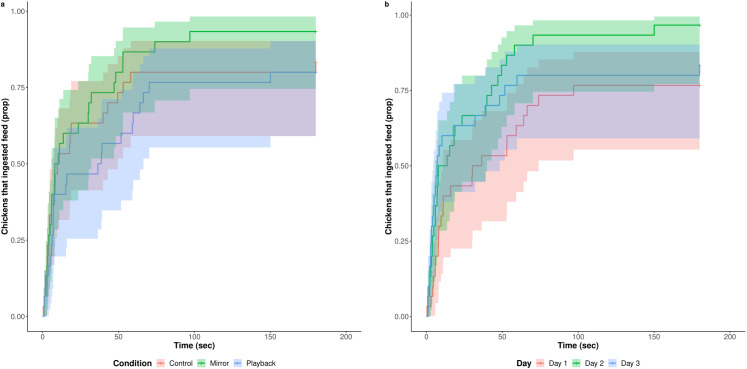
Cumulative incidence of first contact with the feed (as proportion of observations) for chickens in different conditions (a) and on different test days (b, N = 30).

Feed ingestion was observed in 83.3% of tests. There was no difference between conditions (P = 0.215) and test days (P = 0.434) in the likelihood to ingest feed. Average latency to feed was 46.79 seconds. Latency to feed was longer when playback (mean: 60.15 seconds) was presented than when the mirror was presented (mean: 31.61 seconds, HR = 2.569, P = 0.008, Fig 2, S2 Table) and on day 1 (mean: 63.96 seconds) compared to day 2 (mean: 29.52 seconds, HR = 0.236, P <  0.001) and day 3 of testing (mean: 46.88 seconds, HR = 0.24, P <  0.001). No other differences between conditions or days were found (all P >  0.05). The object was touched in 17.8% of tests and mean latency to touch it was 158.92 seconds. Chickens were similarly likely to touch the object in all conditions (P = 0.435) and on all days (P = 0.626). Latency to touch the object was not affected by either condition or test day (χ2 = 2.479, df = 4, P = 0.648, S3 Table). On average, chickens spent 63.52% time feeding. Duration of feeding was similar between conditions and test days (P = 0.271, S4 Table). Exploration of the arena was observed in 32.2% of tests and was equally likely in all conditions (P = 0.765) and on all test days (P = 0.287).

**Fig 3:**
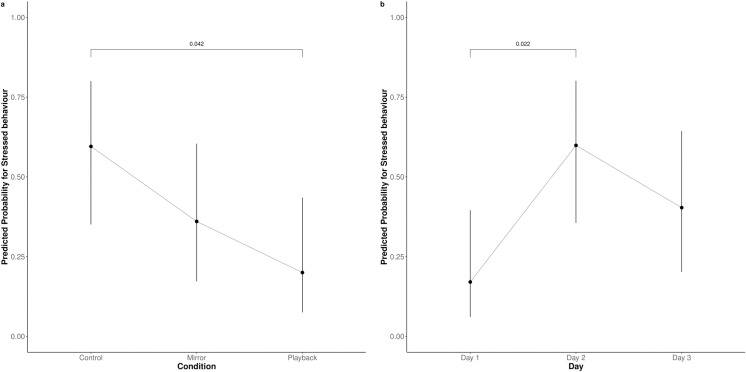
Predicted probability for stress behaviour for chickens in different conditions (a) and on different test days (b, N = 30).

In 51% of tests, chickens exhibited at least 30 seconds of vigilant behaviour, which was not affected by either condition (P = 0.068) or test day (P = 0.572). In 40% of tests, chickens displayed stress behaviour. Chickens were less likely to exhibit stress behaviour when presented with the playback than in the control condition (OR = 0.17, P = 0.042, Fig 3). Although not statistically significant, numerically stress behaviour was also less likely in the mirror than in the control condition. Chickens were less likely to show stress on day 1 than on day 2 of testing (OR = 0.14, P = 0.022). No other conditions or days influenced the likelihood for stress behaviour (P >  0.05).

Irrespective of condition and test day, the maximum eye and comb temperatures decreased after the test (estimated decrease: −0.400° C for eye temperature, P = 0.032, and −2.346 for comb temperature, P = 5.49e-08, respectively; S5 and S6 Tables). Neither the condition nor the test day affected the difference in maximum eye temperature (P = 0.520) or comb temperature (P = 0.643).

## Discussion

The aim of this study was to investigate whether the provision of a (1) mirror or (2) video and sound playback of the conspecifics in the home pen (i.e., playback) could reduce stress in socially isolated adult domestic chickens. The findings confirmed that social isolation elicited a mild stress response in chickens, as evidenced by reduced surface eye and comb temperatures following isolation coupled with the exhibition of vigilance and stress behaviour during isolation. However, most chickens consumed feed during testing, suggesting that the stress response was not severe.

In line with the prediction, chickens were less likely to exhibit stress behaviour when exposed to the playback condition compared to the control. The mirror condition also appeared to reduce the likelihood for stress behaviour compared to the control condition, although this finding was not statistically significant and should therefore be interpreted with caution. Interestingly, the mirror condition resulted in a shorter latency to feed compared to the playback; however, neither condition significantly differed from the control condition in this regard, contrary to predictions. Vigilance behaviour, typically associated with perceived threat [39], and surface temperature reduction were unaffected by condition, implying that only specific stress-related behaviours were influenced.

Exploration behaviour was infrequently observed and appeared unaffected by condition, likely due to the short test duration. In about half of tests, chickens displayed at least 30 s of vigilance behaviour, and in most tests, they spent majority of their time feeding, leaving limited opportunity for other behaviours, such as exploration. Furthermore, the lack of litter in the arena allowed chickens to visually survey their environment without needing to manipulate it. This likely contributed to the limited expression of exploration behaviour.

### Simulated companionship through mirrors and playbacks

In adult chickens, a combination of audio and video playbacks appears to provide a sense of companionship [13]. This is demonstrated by an increase in alarm calls when presented with predator models alongside a playback of another chicken, compared to a playback of an empty cage or a quail [13]. Additionally, video playback presentations of chickens elicit approach responses in both young and adult chickens [12,40]. These observed approach behaviours, combined with the reduction in stress behaviour noted in this study, suggest that video playbacks might have a calming effect on socially isolated chickens, likely by simulating companionship.

Chickens are drawn to their own mirror reflections and approach them [41–43], and young chickens presented with mirrors exhibit a reduction in stress vocalisations during isolation [44], indicating that young chickens likely perceive their own reflection as a companion. In this study, adult chickens also displayed a reduction in stress behaviour and shorter latency to feed in the mirror condition compared to the control condition. However, as these results were not statistically significant, they should be interpreted cautiously.

Interestingly, vigilance behaviour, which is known to decrease when chickens forage in pairs or are tested in arenas with companions compared to isolation [45,46], did not differ between conditions in this study. The lack of physical interaction with companions in both the mirror and playback conditions may explain the partial reduction in stress-related behaviours observed. Studies involving live companions often allow unrestricted physical contact, which provides a more complete social experience [45,46].

A notable difference between this study and previous work is that the chickens in the present study were naïve to the mirrors and playback and not habituated to them. Habituation has been shown to influence how chickens perceive and interact with their environment and stimuli [47]. For instance, Hillemacher et al. (2023) [47] reported that habituated chickens could distinguish their own reflections from the sight of other chickens, leading to distinct behavioural responses to predatory stimuli. In the absence of habituation, it is likely that the chickens in the current study perceived the playback or mirror as novel stimuli, rather than as familiar companions, which may have limited their calming effect on vigilance. However, it is important to consider that habituation to mirrors might alter how chickens perceive their reflection [47]. If chickens recognise the reflection as themselves rather than as a conspecific, the stress-reducing effect of perceived companionship may be diminished. Future longitudinal studies investigating repeated exposure to mirrors and playbacks are recommended to address these questions.

### Emotional contagion in stress reduction

Chickens may experience emotional contagion, particularly in group settings [48,49]. For instance, adult chickens exhibit behavioural and physiological stress responses, such as increased vigilance, heightened vocalisations, and reduced surface temperature, when their own chicks kept in pairs are stressed [50]. Likewise, young chickens also display stress responses when observing groups of stressed conspecifics [48]. However, these responses, indicative of emotional contagion, are absent in adult chickens observing individual familiar adult chickens exposed to the same stressors [51]. As a social species, chickens are likely to respond more readily to cues from several conspecifics, such as those presented in the playback condition of this study, rather than from a single individual.

The effects of emotional contagion on stress reduction remain unclear. While emotional contagion as a response to positive experience has not been studied in chickens, its calming effects have been documented in other non-human species [52]. Thus, it is possible that the playback’s portrayal of a group of calm, contented chickens displaying behaviours associated with neutral or positive affective states (e.g., [53]) could have mitigated stress behaviours through emotional contagion. In contrast, mirrors reflecting vigilance and other stress-induced changes in chicken appearance [54], might not have provided the same effect. Future research should examine whether playback or mirror conditions evoke different degrees of emotional contagion in chickens.

### Social facilitation of feeding

Social facilitation plays a key role in feeding behaviour, as chickens prefer to feed in the presence of companions [55,56]. Likewise, a combination of audio and video presentation of a feeding chicken increases the amount of feed consumed compared to a video playback of an empty cage [57] indicating possible socially facilitated feeding in response to a playback of a feeding conspecific. However, the playback condition in this study did not include visible food, limiting its influence on feeding behaviour. In contrast, in the mirror condition, in the reflection, feed treats from the food tray in front of the mirror were visible and likely contributed to the shorter latency to feed [58,59].

### Study restrictions and recommendations

This study compared the control condition to mirrors and playbacks, but future research should include playbacks without companion chickens to isolate the effects of conspecifics presence. In this study, numerical trends could be observed in the stress behaviour and latency to feed. However, the statistical analysis did not confirm the effect of the condition on these behaviours. Exploring individual personality traits, conducting longitudinal studies, and varying environmental settings could provide additional insights. Future studies should also separately address the auditory and visual components of playbacks and use less palatable feed to examine feeding behaviour more effectively.

## Conclusion

This study indicates that playback and to some extent mirror mitigate stress in socially isolated adult chickens, although not all types of stress-related behaviour, such as vigilance, were influenced. Future research is required to refine these methods and understand their effects on habituation and stress reduction, potentially improving animal welfare and research methodologies.

## Supporting information

S1 Fig Indoor part of the home pen. Left from perched is the feed pan used for social isolation testing left in the home pen full of feed treats for habituation.
(DOCX)

S2 Fig Outdoor wintergarden and test arena. (DOCX)

S3 Fig Test arena setup. Behind the mirror the computer screen was positioned. Depending on the experimental condition, the chickens were presented with either a computer screen, front, or back side of the mirror. The position of the food tray remained the same across all three experimental conditions.
(DOCX)

S1 Table Results of the intra- and inter-rater reliability testing. (DOCX)

S2 Table Latency to feed (s). (DOCX)

S3 Table Latency to touch object (s). (DOCX)

S4 Table Feeding duration (s). (DOCX)

S5 Table Mean of the maximum comb temperature (°C) across conditions and days. (DOCX)

S6 Table Mean of the maximum eye temperature (°C) across conditions and days. (DOCX)

S1 Data Data-behaviour. (CSV)

S2 Data Data-surface-temp. (CSV)
