# Supplementary material for: Alleviating isolation stress in chickens: The benefits of home pen playback and mirrors
Source: PLoS One. 2025 Feb 12;20(2):e0318126. doi: 10.1371/journal.pone.0318126 (PMC11819468; doi:10.1371/journal.pone.0318126)
Supplement: S1 Fig — Left from perched is the feed pan used for social isolation testing left in the home pen full of feed treats for habituation. (DOCX) [file pone.0318126.s001.docx]

Alleviating Isolation Stress in Chickens: The Benefits of Home Pen Playback and Mirrors

Janja Sirovnik

Centre for Animal Nutrition and Welfare, Clinical Department for Farm Animals and Safety of Food Systems, University of Veterinary Medicine, Vienna, Austria

janja.sirovnik-koscica@vetmeduni.ac.at


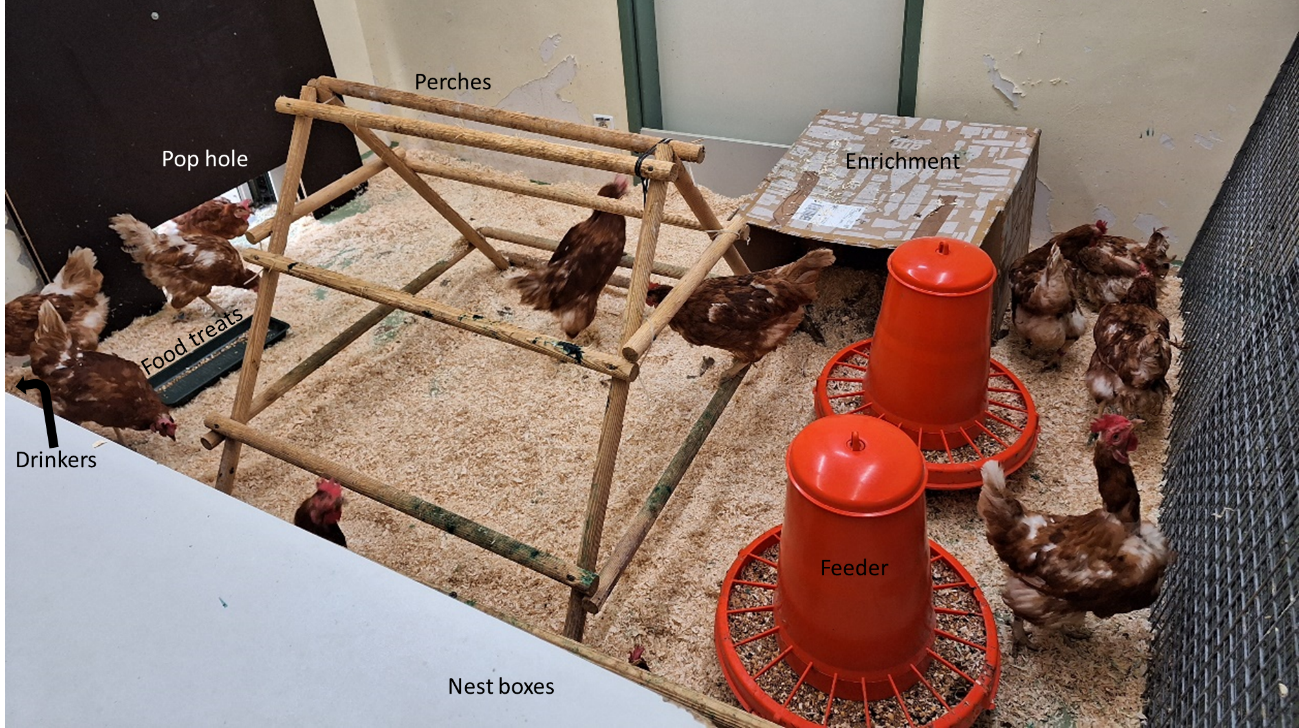


**S 1 Fig. Indoor part of the home pen. Left from perched is the feed pan used for social isolation testing left in the home pen full of feed treats for habituation).**
