# Supplementary material for: Alleviating isolation stress in chickens: The benefits of home pen playback and mirrors
Source: PLoS One. 2025 Feb 12;20(2):e0318126. doi: 10.1371/journal.pone.0318126 (PMC11819468; doi:10.1371/journal.pone.0318126)
Supplement: S3 Fig — Behind the mirror the computer screen was positioned. Depending on the experimental condition, the chickens were presented with either a computer screen, front, or back side of the mirror. The position of the food tray remained the same across all three experimental conditions. (DOCX) [file pone.0318126.s003.docx]

Alleviating Isolation Stress in Chickens: The Benefits of Home Pen Playback and Mirrors

Janja Sirovnik

Centre for Animal Nutrition and Welfare, Clinical Department for Farm Animals and Safety of Food Systems, University of Veterinary Medicine, Vienna, Austria

janja.sirovnik-koscica@vetmeduni.ac.at

# Supplementary material


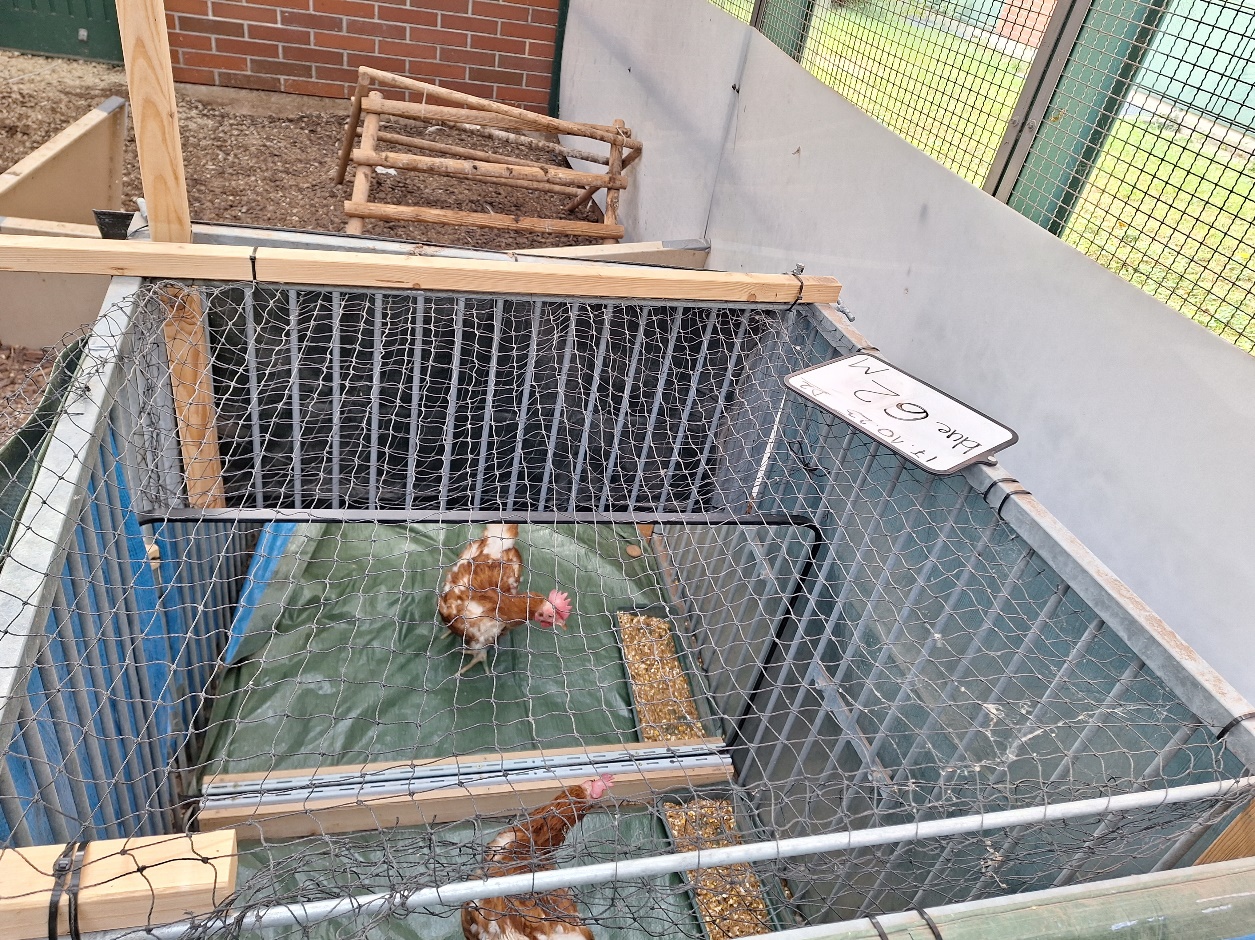


**Figure S 3. Test arena setup. Behind the mirror the computer screen was positioned. Depending on the experimental condition, the chickens were presented with either a computer screen, front, or back side of the mirror. The position of the food tray remained the same across all three experimental conditions.**
