# Supplementary material for: Alleviating isolation stress in chickens: The benefits of home pen playback and mirrors
Source: PLoS One. 2025 Feb 12;20(2):e0318126. doi: 10.1371/journal.pone.0318126 (PMC11819468; doi:10.1371/journal.pone.0318126)
Supplement: S5 Table — (DOCX) [file pone.0318126.s008.docx]

Alleviating Isolation Stress in Chickens: The Benefits of Home Pen Playback and Mirrors

Janja Sirovnik

Centre for Animal Nutrition and Welfare, Clinical Department for Farm Animals and Safety of Food Systems, University of Veterinary Medicine, Vienna, Austria

janja.sirovnik-koscica@vetmeduni.ac.at

# Supplementary material

**Table S 5. Mean of the maximum comb temperature (^◦^C) across conditions and days.**

|  | **Mean of max T** | **SD of max T** |
| --- | --- | --- |
| **Condition** |  |  |
| Control | **30.91** | **3.18** |
| after | 29.51 | 3.06 |
| before | 32.36 | 2.65 |
| Mirror | **30.67** | **3.41** |
| after | 29.46 | 3.22 |
| before | 31.87 | 3.21 |
| Playback | **31.67** | **2.84** |
| after | 30.67 | 2.12 |
| before | 32.67 | 3.13 |
| **Day** |  |  |
| Day1 | 31.35 | 2.99 |
| after | 30.30 | 2.68 |
| before | 32.40 | 2.96 |
| Day2 | 30.96 | 3.05 |
| after | 29.89 | 2.99 |
| before | 32.03 | 2.76 |
| Day3 | 30.93 | 3.47 |
| after | 29.44 | 2.95 |
| before | 32.48 | 3.33 |
| **Mean T after** | **29.88** | **2.86** |
| **Mean T before** | **32.30** | **3.00** |
